# Techniques and Strategies to Minimize Radiation Exposure in Pediatric Computed Tomography (CT) Abdominal Examinations: A Review

**DOI:** 10.7759/cureus.67494

**Published:** 2024-08-22

**Authors:** Inayatullah Shah Sayed, Muhammad Irfan Mohd Yusof

**Affiliations:** 1 Department of Diagnostic Imaging and Radiotherapy, Kulliyyah of Allied Health Sciences, International Islamic University Malaysia, Kuantan, MYS

**Keywords:** abdominal ct, alara, radiation-induced malignancies, pediatric, radiation protection

## Abstract

As children are more vulnerable to radiation-induced cancers and have longer life expectancies, it is essential to implement strict radiation protection measures in pediatric imaging. This study aimed to review radiation dose-minimizing measures in pediatric abdominal computed tomography (CT) examinations. A systematic search across various databases, including Web of Science, PubMed, SpringerLink, ScienceDirect, and Google Scholar, yielded a total of 7,314 articles. The search used keywords that aligned with the objectives of the study. This study included 77 publications after applying the criteria for inclusion and exclusion. We carefully reviewed these selected articles for compliance with the inclusion criteria and excluded them if they did not meet the specified criteria. Only 12 articles fulfilled the strict criteria. An in-depth review of 12 selected articles demonstrated the radiation dose reduction techniques and strategies, which include prefiltering and post-processing algorithms, careful adjustment of exposure parameters such as tube voltage (kVp) and current (mAs), and the establishment of diagnostic reference levels (DRL). Reduction of radiation exposure in pediatric CT imaging demands multifaceted approaches. To reduce the ionizing radiation dose while still obtaining high-quality diagnostic images, healthcare practitioners should adhere to DRL, adjust exposure factors, implement prefiltration, employ AI, and use post-processing algorithms.

## Introduction and background

Computed tomography (CT), including multi-detector computed tomography (MDCT) scans, has revolutionized medical imaging by offering necessary diagnostic information for a wide range of medical disorders. CT scans are essential for identifying many injuries and traumas because they have the ability to accurately visualize fresh bleeding, bone fractures, aortic damage, diaphragmatic rips, fractures, and abdominal trauma, such as a ruptured spleen [1]. The utilization of CT for investigating the health conditions in pediatric patients has witnessed an increase by a factor of 8 in recent years as compared to the 1980s. This trend is expected to persist at an annual growth rate of 10% [2]. Although CT scans provide valuable diagnostic capabilities, it is important to consider the possible risks associated with ionizing radiation, particularly when dealing with pediatric patients. This is necessary in order to maintain a balance between the diagnostic benefits and the risk of radiation-induced malignancies [3].

As documented in a study by Obara et al. [4], radiation exposure to young infants is relatively low; however, it does have biological effects. The reported assessed effective doses for the abdominal region in zero-, one-, and five-year-old patients were 2.83±3.0, 3.12±2.3, and 1.91±1.7 mSv, respectively. Ionizing radiation dose can cause two types of biological consequences, e.g., deterministic effects and stochastic effects. Pediatric patients are more prone to developing radiation-induced malignancies due to their increased radio-sensitivity to ionizing radiation [5].

Stochastic effects are further classified into somatic and genetic effects, and they are characterized by the absence of a threshold dose. As radiation exposure increases, there is a corresponding rise in the probability of acquiring cancer disease. If, on the other hand, the radiation dose is more beyond the threshold for deterministic effects, then complications may occur. Erythema, sterility, and cataracts are a few examples of the many potential adverse consequences that might occur [6].

Strategies for reducing radiation dose in pediatric CT examinations

Ionizing radiation exposures can be reduced by adjusting several variables in pediatric CT abdominal examinations. Common CT procedures optimize scan length, tube current, tube voltage, pitch, beam collimation, and slice width for each patient to shorten scan durations [7,8]. Healthcare facilities may achieve the adjustment of these factors in different ways depending on the operator. The implementation of the "as low as reasonably achievable" (ALARA) principle directs these endeavors.

Psychological Considerations

An environment characterized by calmness and serenity is crucial for obtaining high-quality CT images in pediatric patients. In order to foster psychological calm in children, it is imperative that the CT room is tranquil and tailored to their specific requirements. In addition, maintaining a child's room at a reasonable temperature and integrating entertaining drawings and pictures can assist in reducing their anxiety [9]. Prioritizing the establishment of a conducive atmosphere prior to the clinical examination significantly enhances the probability of achieving success [10]. For pediatric patients, distraction strategies and techniques must be considered. Moreover, pediatric patient-centered CT rooms and familiarity with the surroundings all contribute to ensuring patients stay calm and cooperative. Effective communication with pediatric patients is indispensable. In addition, it is important to provide clear and explicit instructions about patient positioning, breathing exercises, and continuous monitoring during the CT imaging procedures. Efficient communication reduces the probability of needing to retake an examination and limits the chance of mistakes. Respiration exercises can enhance the effectiveness of an examination when deemed appropriate [11]. Proper positioning of the patient is essential to achieve effective radiation protection. Isocenter placement is necessary for automated exposure control to adjust the radiation dosage based on the patient's size. To provide accurate results and minimize the occurrence of repeated scans and motion artifacts, it is crucial to limit patient movement throughout the examination. One method to immobilize pediatric patients is by securely tying their legs [9,10].

Technical Considerations

It is important to remember that technological considerations greatly contribute to reducing ionizing radiation exposure to patients. The association between the radiation dosage and CT scan pitch is inversely proportional. Although raising the pitch can reduce radiation exposure, it can also impair image quality. In order to strike a balance between the quality of the image and the amount of radiation exposure, it is recommended to keep the pitch between the range of 1 and 1.5 [10,12]. Correctly aligning the patient according to measurements reduces radiation exposure. Collimators absorb main beam radiation regardless of arrangement. Patient size, noise, 3D reformatting, and low radiation exposure should be considered while modifying collimation [13]. Modern scanners can lower radiation by 38% using adaptive section collimation [14].

Prefiltration in CT Imaging

Current objectives in medical imaging are to reduce radiation doses and increase diagnostic accuracy. Additional prefilters improve the efficacy of CT imaging in healthcare. This technique has improved patient safety by decreasing radiation exposure while adhering to the ALARA concept. CT imaging requires prefiltration to insert several prefilters and selectively filter the X-ray beam. The use of selective filtration suppresses the transmission of low-energy photons and enhances the penetration of high-energy photons, resulting in a reduction in the patient's radiation dose. This procedure significantly reduces patient radiation exposure during clinical testing while generating high-quality diagnostic images.

Advances in technology also influence healthcare practices, specifically in terms of patient protection and ALARA principles. According to the ALARA principles, diagnostic techniques have greatly reduced radiation exposure. This ideology stresses minimizing radiation exposure to the lowest feasible levels while simultaneously improving patient safety. Moreover, medical imaging professionals are now able to provide high-quality diagnostic images while simultaneously minimizing radiation exposure to patients.

To optimize the contrast-to-noise ratios while minimizing the radiation exposure, there are some effective approaches, such as reducing tube voltages and using prefilters. These approaches significantly reduce radiation dose to patients during diagnostic imaging procedures, which is a major concern among patients and the medical imaging community.

The abovementioned developments demonstrate the commitment of the medical community to guaranteeing patient safety and laying the foundation for future advancements in the realm of medical imaging. The incorporation of prefilters into CT imaging indicates a significant advancement in the field of medical imaging technology. This strategy successfully reduces the primary danger of radiation exposure while also improving the accuracy of diagnosis. Innovative methods are currently strengthening the commitment to the protection of patients, as demonstrated by the ALARA principle, and setting a path towards a future that strikes the right balance between the risks of radiation and diagnosis accuracy. The field of radiology is poised to see significant improvements and transformative changes as a result of the continuous growth in medical imaging equipment.

ALARA

To decrease radiation exposure in children in CT imaging, implementation of the ALARA principle is a must. With ALARA, one can limit, optimize, and defend the dosage. In medical imaging with ionizing radiation, the objective is to provide precise diagnostic data while reducing radiation exposure to the minimum. Particularly for younger patients who are more susceptible to radiation's adverse effects, this is essential for lowering radiation risks [7,15]. Hence, since the implementation of the ALARA principle in the 1960s [14], the primary objective has been to ensure minimum pediatric patient doses in CT imaging. These regulations guarantee the safety, efficacy, and low radiation exposure of CT scans for children.

Artificial intelligence (AI) in post-processing for dose reduction in pediatric CT examinations

For reducing the radiation dose to pediatric patients in CT clinical examinations, there is an increasing trend of applying AI in post-processing. Research published by Zhang et al. [16] presented the capability of convolutional neural networks (CNN) to decrease radiation exposure by 36-70% while maintaining outstanding diagnostic image quality. Four studies utilizing pediatric abdomen CT images provide evidence for the efficacy of dose reduction through the application of deep learning image reconstruction (DLIR) algorithms. Zhang et al. [16] found that deep learning image reconstruction-high (DLIR-H) decreased radiation dose by 70% compared to previous reconstruction approaches. Kim et al. [17] and Brady et al. [18] both saw a significant reduction in radiation exposure following the use of DLIR. Nagayama et al. [19] found that DLIR results in a 50% decrease in radiation dose compared to hybrid iterative reconstruction (HIR), as per their research. It is necessary to optimize radiation doses in CT imaging to ensure the ionizing radiation safety of pediatric patients during clinical examinations. A review article by Zhang and Seeram [20] highlights a diverse array of methods and strategies, including the application of DLIR, optimization of exposure parameters, prefiltering, and AI post-processing. By effectively implementing these principles, it is likely to achieve a significant decrease in radiation dose without compromising the diagnostic effectiveness of CT scans. By using these approaches, medical practitioners can minimize the radiation exposure of young children while maintaining the accuracy of their diagnosis.

This study aimed to provide knowledge on the current best practices for radiation protection during CT abdominal imaging in children. Further, this review study conducted a thorough search across various reputable sources, including PubMed, Google Scholar, SpringerLink, and ScienceDirect, to retrieve the published research articles that match the objectives of the study. Specific criteria for inclusion or exclusion were set for the selection of articles.

## Review

Methods

A systematic search across different databases to collect articles relevant to the objectives of the study was undertaken. The relevant information from the selected articles was extracted, the collected data was organized, and information was analyzed according to thematic categories.

Search of Articles

The search process for retrieving the articles relevant to the study's objectives was initiated by identifying different databases, including Google Scholar, PubMed, SpringerLink, and ScienceDirect. "Computed tomography," "pediatric patients," "abdomen," "optimum amount of radiation exposure," and "radiation protection" were the keywords. In addition, synonyms and related terms were applied interchangeably, e.g., "children" for "pediatric" and "dose reduction" for "radiation reduction." Boolean operators (AND, OR) were employed to refine search results. For example, a sample search statement was ((radiation protection) OR (radiation reduction)) AND ((MDCT) OR (computed tomography) OR (CT)) AND (abdomen OR abdominal) AND (pediatric).

Different filters as per database options were used to augment the specificity. For instance, filters included a range of publication years (2019-2023) and text availability (e.g., "open access") where applicable. Moreover, article type filters like a "review article" and "research article" were applied in ScienceDirect, Google Scholar, and SpringerLink.

Criteria for the Selection of Articles

The process of article selection adhered to predetermined inclusion and exclusion criteria, as described in Table 1. As per the criteria, only studies involving pediatric patients, undergoing computed tomography scans of the abdomen region, and using CT were included in the analysis.

**Table 1:** Inclusion and exclusion criteria for article selection.

Inclusion criteria	Exclusion criteria
Studies involving pediatric patients	Studies limited to adult and geriatric patients
Studies covering the abdomen region	Studies not related to the abdomen region
Studies utilizing computed tomography	Studies using first- to third-generation and dual-source computed tomography

Screening of Articles

**Figure 1:**
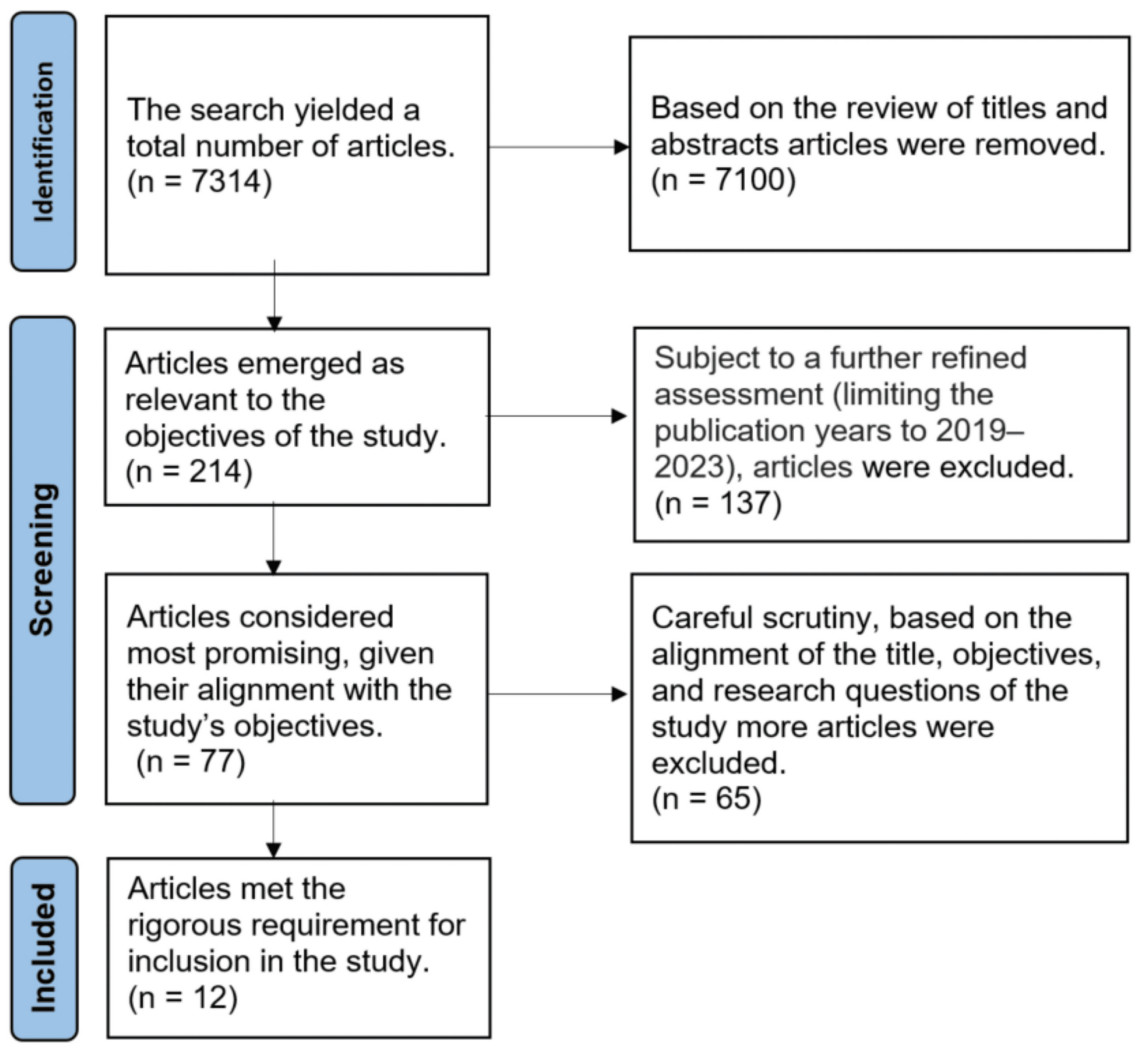
Flowchart showing the selection process of articles.

All articles gathered from the databases went through a screening process based on their titles and abstracts to classify those most pertinent to the study. Additional screening was conducted on chosen articles to discard duplicates, and only articles with full text were assessed. After that, the selected articles underwent a detailed review to ensure they met the inclusion criteria and excluded those that did not. Figure 1 shows the process for selecting articles according to the Preferred Reporting Items for Systematic Reviews and Meta-Analyses (PRISMA) guidelines.

Organization of Selected Articles

Selected articles were organized by documenting details and relevant information according to the identified themes. These themes were classified based on the content of the reviewed articles. Also included were the title, the year of publication, the authors, the theme, and the gathered information. This procedure summarizes the systematic approach taken to categorize and select relevant articles, ensuring the quality and relevance of the included studies in the review.

Results

As the result of scrutiny of a total of 7,314 articles, 214 articles were identified which were relevant to the study's aims. Further, a systematic evaluation was carried out on these 214 articles. For this step, other criteria such as limiting the publishing years to 2019-2023 and checking for repeated titles were included. Following the adoption of these rigorous criteria, a total of 77 articles were left for further evaluation. All 77 publications were downloaded as a full paper. Another crucial step in the selection process was a thorough analysis of the whole contents of the 77 publications. Every item was subjected to a thorough analysis procedure to determine its conformity with the study queries, objectives, and title of the inquiry. The use of specific inclusion and exclusion criteria helped to identify articles that directly addressed the aims and objectives of the current investigation. Finally, only 12 articles met the rigorous requirements for inclusion in the study (Table 2). These articles were chosen for their relevance and content quality, suggesting that they possessed the most significant information needed to contribute meritoriously to the study's objectives.

**Table 2:** Summary of articles that met the predefined inclusion criteria. CT: computed tomography; DLIR: deep learning image reconstruction; DLR: deep learning reconstruction; IR: iterative reconstruction; AD: achievable dose; ATCM: automatic tube current modulation; AI: artificial intelligence; CD: Crohn's disease; RD-CTE: reduced-dose computed tomography enterography; MBIR: model-based iterative reconstruction; SD-CTE: standard-dose computed tomography enterography; AEC: automatic exposure control; ASIR: adaptive statistical iterative reconstruction; CNR: contrast-to-noise ratio

Author(s)	Title	Theme(s)	Point
Zhang et al. (2022) [16]	Deep learning image reconstruction in pediatric abdominal and chest computed tomography: a comparison of image quality and radiation dose	Post-processing	The use of DLIR enables the reduction of radiation doses in pediatric CT scans without compromising image quality
Kim et al. (2023) [17]	Effect of tube voltage and radiation dose on image quality in pediatric abdominal CT using deep learning reconstruction: a phantom study	Post-processing and parameter setting	The utilization of DLR and 100 kVp is anticipated to be employed for the purpose of ensuring the acquisition of safe clinical images in the pediatric population
Brady et al. (2021) [18]	Improving image quality and reducing radiation dose for pediatric CT by using deep learning reconstruction	Post-processing	Radiation dose reduction and image quality enhancement were achieved with the dose-limiting reconstruction technique while maintaining the noise texture and spatial resolution integrity
Nagayama et al. (2021) [19]	Deep learning-based reconstruction for lower-dose pediatric CT: technical principles, image characteristics, and clinical implementations	Post-processing	A substantial dose reduction in abdominal CT with high image contrast is possible by applying IR methods
Satharasinghe et al. (2022) [21]	Patient size as a parameter for determining diagnostic reference levels for paediatric computed tomography (CT) procedures	Establishment of DRL	AD values have the potential to reduce doses by up to 90% when used as a baseline for target dosage optimizations
Papadakis and Damilakis (2019) [22]	Automatic tube current modulation and tube voltage selection in pediatric computed tomography	Parameter setting	ATCM decreases radiation dosage while maintaining image noise while reducing radiation dosage by 41% and 48%, respectively, for the abdomen and pelvis
Ng (2022) [23]	Artificial intelligence for radiation dose optimization in pediatric radiology: a systematic review	Post-processing	The majority of research showed that radiation doses might be lowered by 36-70% using AI while maintaining diagnostic information
Lee et al. (2022) [24]	Image quality and diagnostic accuracy of reduced‑dose computed tomography enterography with model‑based iterative reconstruction in pediatric Crohn's disease patients	Post-processing and parameter setting	When identifying the presence of active CD in the terminal ileum of the small bowel, RD-CTE utilizing 80 kVp in conjunction with MBIR reduced the dose by more than 80% compared to SD-CTE while ensuring similar diagnostic precision
Ahmed et al. (2021) [25]	Multi‑detector computed tomography in traumatic abdominal lesions: value and radiation control	Parameter setting	The AEC technique is a highly significant method for reducing doses, particularly in children, by approximately 30-50%
Mokhtar et al. (2021) [26]	Studies on the radiation dose, image quality and low contrast detectability from MSCT abdomen by using low tube voltage	Parameter setting	The radiation dosage recorded at 80 kVp and 380 mAs was 37% lower than the dose measured at 120 kVp and 300 mAs. Therefore, it is believed that pediatric abdominal CT scanning at 80 kVp is possible without compromising diagnostic accuracy when mAs exceeds 300 mAs, thereby permitting a reduction in radiation dose of 29-37%
Steidel et al. (2022) [27]	Dose reduction potential in diagnostic single energy CT through patient-specific prefilters and a wider range of tube voltages	Parameter setting and filtration	Significant reductions in dosage can be attained with the implementation of patient-specific prefiltration techniques and the utilization of tube voltages below 70 kVp
Sun et al. (2020) [28]	Performance evaluation of two iterative reconstruction algorithms, MBIR and ASIR, in low radiation dose and low contrast dose abdominal CT in children	Post-processing and parameter setting	MBIR and ASIR effectively mitigate the risks associated with ionization and contrast agent-induced harm. Furthermore, the utilization of MBIR and ASIR enhances the CNR of the images and results in the production of high-quality diagnostic images

The selected articles predominantly focused on pediatric patients and phantoms simulating pediatric patients. These studies encompassed a broad age range, from infants to 16-year-olds. The subsequent data analysis unveiled several common themes that were central to the field of pediatric radiography. These themes included the establishment of dose reference levels (DRL), post-processing techniques, filtration methods, and parameter settings.

Establishment of DRL

The establishment of DRL is a remarkable approach that has appealed to the attention of academia. Satharasinghe et al. [21] utilized the attainable dose (AD) and DRL as baselines and reported a reduction of radiation exposure up to 90%. Muhammad et al. [29] emphasize the need for applying DRL to make the best use of it for radiation exposure minimization in CT scans of pediatric patients.

Technical Parameter Setting

When it comes to optimizing radiation doses in pediatric CT abdominal examinations, technical parameter setup is crucial. Several research articles have reported the optimization of technical factors for radiation dose reduction. According to Kim et al. [17], the square of the tube voltage is directly proportional to the radiation dosage. They argued for using low tube voltage and identified 100 kVp as an optimal and safe radiation dosage for pediatric abdominal imaging. The results of Mokhtar et al. [26], who used 80 kVp instead of the conventional 120 kVp, show that the dosage is reduced by 63%. Ahmed et al. [25] highlight the benefits of AEC in modulating exposure factors based on patient size, achieving up to 50% dose reduction in pediatric abdominal CT examinations.

Automatic Tube Current Modulation (ATCM) and Automatic Tube Voltage Selection (ATVS)

Papadakis and Damilakis [22] show that ATCM and ATVS can cut radiation dose by a large amount, though the amount of reduction varies by age group and clinical mode.

Prefiltration

The application of prefilters, in conjunction with low tube voltages, is an effective approach for minimizing radiation doses in CT examinations. Steidel et al. [27] investigated by using tin and copper prefilters with varied thicknesses and tube voltages. They demonstrated radiation dose reductions of up to 67% for specific configurations, making prefilters a promising method for pediatric CT dose optimization.

Post-processing

An increasing interest in applying AI for post-processing has been witnessed in order to minimize the radiation dose to patients in diagnostic radiology. In 2022, a study conducted by Ng [23] showed that CNN reduced radiation exposure by 36-70% while maintaining high-quality diagnostic images. A 70% reduction in radiation dose with the use of the DLIR-H technique was achieved as compared to the previous technique [16]. Moreover, by applying the DLIR technique, Brady et al. [18] and Kim et al. [17] have reported in their research work a significant reduction in radiation exposure. In their study, Nagayama et al. [21] investigated that DLIR leads to a 50% reduction in dose compared to HIR. To ensure the radiation safety of patients during abdominal examinations in pediatric CT imaging, it is indispensable to optimize radiation dosages. Several approaches and strategies for the reduction of radiation exposure to patients in diagnostic radiology exist. The requirements include the use of DLIR, optimization of technical parameters, prefiltering, and AI with post-processing. When these techniques and strategies are meticulously implemented, it is possible to achieve a significant reduction in radiation dose without compromising the diagnostic quality of CT images.

Discussion

CT plays a significant role in the diagnosis, treatment, and management of various human health conditions. However, ionizing radiation can be potentially harmful; therefore, it is imperative to implement techniques and employ appropriate approaches to reduce radiation doses, particularly for pediatric patients. Based on the analysis of extracted information from the reviewed studies, this section discusses various techniques and strategies for minimizing radiation exposure to pediatric patients during CT abdominal imaging.

Establishment of DRL for Minimizing the Radiation Dose

DRL are important for regulating radiation exposure in pediatric CT scanning. According to Satharasinghe et al. [21], DRL for pediatric patients may not be accurate since they are set based only on their age, without taking into account variations in body size. DRL are divided into four groups, 0-1, 1-5, 5-10, and 10-15 years, based on the age of children. The size-specific dose estimate (SSDE) procedure is an additional significant option. The SSDE is based on the effective diameter (Deff), the anteroposterior and lateral diameters (AP and LAT), and patient-specific aspects like age, weight, and body mass index (BMI). This process is fundamental for ensuring that radiation doses are accurately measured to the particular patient's size rather than being merely age-dependent.

DRL are important for radiation safety rules for radiological operations. They can be used in local institutes, imaging facilities, certain regions, or even whole countries. As a result, they allow for the regulation of radiation safety in pediatric patients from radiation dose ensuring that unnecessary exposure is minimized. Furthermore, DRL serve as reference values for radiation doses. This lets healthcare facilities compare their own amounts of exposure to established standards. This facilitates benchmarking, training, ensuring quality, evaluating performance, and improving pediatric CT imaging [28].

Exposure Factor Adjustment for Radiation Dose Reduction

Several factors, such as tube voltage (kVp), tube current (mAs), and automatic exposure control (AEC), can be altered in pediatric CT imaging to lessen the radiation dose.

Tube voltage (kVp): To minimize the radiation exposure, the tube voltage can be lowered. The recommended tube voltage is 80-100 kVp for pediatric patients. AI methods used for post-processing were found to be able to lower tube voltage to 50 kVp. These methods successfully reduced the noise and improved edges, which resulted in better image quality. AI algorithms can be used to optimize acquisition parameters, monitoring dosages, and compliance with guidelines [17].

ATVS: For X-ray images, the best voltage is found automatically through a method called ATVS. ATVS uses modern technology to make sure that the tube voltage is adjusted appropriately during a scanning, taking into account the patient's anatomy and the attenuation factors. By implementing this method, radiation exposure can be minimized without degrading the quality of images. When ATVS is used with other dose reduction techniques, like ATCM and iterative reconstruction algorithms, it makes pediatric CT images better while also minimizing the dose.

Tube current (mAs): If the tube current (mAs) is decreased, it directly decreases the radiation dose, since the current controls the intensity of the X-ray beam. ATCM controls the tube current based on the size of the patient and attenuation. By using this method, the appropriate radiation delivery to all parts of the body is guaranteed, and high-quality images are also made [18].

Prefiltration in CT Imaging for Radiation Dose Reduction in Pediatric Abdominal Scans

CT imaging plays a crucial role in diagnosing various medical conditions, by providing detailed images of internal structures. However, concerns about radiation exposure, especially in pediatric patients undergoing abdominal CT scans, have led to the development and implementation of techniques such as prefiltration.

The prefiltration process: Prefiltration involves the addition of filters along the X-ray beam's path before it reaches the patient. The primary objective is to selectively permit higher-energy X-rays and filter out lower-energy X-rays that contribute to increased patient radiation exposure [30,31]. This filtration process ensures that diagnostic information is not compromised while effectively reducing the overall radiation dose administered to pediatric patients.

Several patient studies have been conducted to examine the potential for dose reduction through the use of additional tin filters. These filters are available in several systems and can be used within the range of 100-150 kVp. Leyendecker et al. [32] successfully achieved a significant 81% reduction in dose for contrast-enhanced abdominal CT. According to Weis et al. [33], a significant dose reduction of 77% was observed in pediatric chest CT scans. Dewes et al. attained a 22% dose reduction in abdominal scans for urinary stone detection. In a study led by Mozaffary et al. [34], it was witnessed that CT scans for urinary stone disease resulted in a dose reduction ranging from 28% to 66%.

Enhancing image quality: For the selection of materials and adjusting the thickness of filters, the CT system's specific characteristics and preset imaging requirements are considered. This personalized methodology improves the balance between dose reduction and image quality. The chosen materials and thicknesses filter out low-energy X-rays, permitting higher-energy X-rays to pass through, resulting in images of higher quality related to those generated without prefiltration. The study of Greffier et al. [35] found that the employment of the tin filter on the Siemens SOMATOM Force system led to improvements in image quality pertaining to lytic and sclerotic bone lesions, pulmonary ground-glass opacity, and the identification of high contrast pulmonary lesions. The main benefit of increasing prefiltration is lowering the dose; however, enhancement in image quality is more valuable in certain applications. Depending on the patient size and contrast agent (soft tissue or iodine enhancement), the radiation dose can be reduced [21].

ALARA principle: Prefiltration is employed in radiological imaging to adhere to the ALARA concept, to reduce radiation dose to patients while preserving the diagnostic accuracy of medical images. The notion of ALARA is a guiding standard in diagnostic radiology for the radiation protection of patients. It allows healthcare professionals to strike a balance between reducing the radiation dose to patients and maintaining high-quality medical images.

Customization for optimal results: Selecting prefiltration materials and thicknesses that match the CT system's demands and imaging capabilities is crucial. A customized filtering method can improve image quality and reduce radiation. The emphasis on customization shows a dedication to medical precision and patient safety.

The primary advantage of supplementary prefiltration is the reduction of dose. Nevertheless, it is essential to highlight that certain applications may get even greater benefits from the improved image quality resulting from the addition of supplementary prefiltration. Furthermore, the incorporation of supplementary prefiltration and other techniques and various strategies can significantly reduce radiation exposure to patients. These techniques include, but are not limited to, iterative reconstruction, personalized filters tailored to individual patients, and control of tube current. The research conducted by Steidel et al. in 2022 [27] has shown that the most optimal outcomes are achieved by integrating both strategies. By utilizing patient-specific prefiltration and tube voltages below 70 kVp, there has been a notable reduction in radiation exposure. The aforementioned comprehensive strategy displays a commitment to improving patient safety and elevating the quality of diagnostic imaging. Prefiltration works best when materials and sizes match the CT system's image criteria. Prefiltration's adjustable capability reduces radiation and improves image quality. This method emphasizes accuracy and patient health.

In conclusion, prefiltration advances pediatric abdominal CT imaging procedures. It systematically reduces radiation exposure while maintaining high-quality medical images. The ALARA concept promotes careful prefiltration material and size selection. This emphasizes patient safety and medical imaging ethics. Modern radiology uses prefiltration to find the greatest balance between radiation reduction and diagnostic accuracy.

Post-processing Algorithms for Radiation Dose Reduction

Post-processing procedures do not directly cut the radiation dose, but they play a vital role in enhancing image quality. CNN are progressively used in post-processing to denoise images acquired at lower radiation doses, decrease artifacts, and improve image quality [17]. Iterative reconstruction methods, for example, iterative image reconstruction algorithm (ADMIRE), adaptive iterative dose reduction (AIDR), and adaptive statistical iterative reconstruction (ASIR), are used to reduce image noise and compensate the degradation in image quality caused by lower tube voltage. The integration of AI into post-processing procedures further improves the efficacy of these algorithms. Furthermore, AI can assist in image denoising, artifact reduction, segmentation, and image enhancement, eventually decreasing the need for higher radiation doses or repeat scans. However, it is imperative to be attentive to increased computational burdens and likely distorted image textures [23].

Benefits of AI in Post-processing for Dose Reduction in CT Scans

Significant dose reduction: A significant advantage of applying AI in post-processing is that it markedly reduces the amount of radiation dose that patients are exposed to during CT scans. Several researches show how well AI, explicitly CNN and DLIR, can reduce the radiation dose ranging from 36% to as high as 70% [23].

Preservation of diagnostic image quality: Studies have shown that AI-based post-processing methods, like DLIR, may keep or improve the quality of diagnostic images even when they are made with smaller amounts of radiation. This is essential in ensuring that medical professionals can still obtain accurate and reliable information from the CT images, contributing to effective diagnosis and treatment planning.

Improved safety for pediatric patients: Radiation dose reduction in pediatric CT imaging is exclusively vital because lowering radiation doses in this age group lowers the chance of long-term health problems associated with radiation exposure. AI plays an important role in enhancing the safety of pediatric patients undergoing CT imaging for the investigations of health conditions.

Enhanced efficiency and workflow: The use of AI in the field of medical imaging improves efficiency and efficacy by quickly processing and reconstructing images compared to conventional approaches. Subsequently, improvement in productivity is attained in healthcare settings. This, in turn, allows healthcare practitioners to make more quickly their decisions relating to the diagnosis and treatment of disorders.

Challenges of AI in Post-processing for Dose Reduction in CT Scans

Development and intricacy of algorithms: Designing and training AI algorithms may be challenging, mainly complex ones like CNN. For writing robust and reliable algorithms, extensive knowledge and computing resources are required. Therefore, these algorithms can effectively cater to various patient populations and imaging disorders.

Diversity and quality of the data: The performance of AI techniques is considerably affected by large and high-quality training data. In terms of guaranteeing the applicability of algorithms in practical circumstances, it is imperative to employ diverse datasets that include a wide range of patient characteristics, anatomical variances, and imaging conditions.

Ethical and regulatory issues: In diagnostic imaging, ethical and legal issues about the use of AI exist, such as adherence to regulatory standards, data privacy issues, and developing standards for the appropriate use of AI in the healthcare sector.

Training and user acceptance: Healthcare practitioners may require specialized training before they can effectively utilize the post-processing technologies driven by AI. Moreover, for optimal use, it is imperative to ensure user compliance and provide proper training.

Expenses and allocation of resources: In the process of AI implementation in healthcare, initial costs incur, such as acquiring the right technology, guaranteeing their impeccable integration with current systems, and providing comprehensive training. During the post-processing phase, healthcare providers may apply AI to identify the most cost-effective processes and allocate resources judiciously, leading to low radiation doses to patients.

To mitigate radiation-associated risks for pediatric patients in CT imaging and provide accurate and valuable diagnoses, the literature emphasizes the need to implement an all-inclusive approach that integrates contemporary technology, such as AI-driven post-processing, with traditional approaches. This advancement indicates a noteworthy achievement in the endeavors to improve patient radiation safety in CT imaging of young patients.

## Conclusions

Systematic and careful analysis of the published literature collected in this review study showed the significance of different techniques and strategies of decreasing ionizing radiation dose in pediatric CT abdominal imaging while preserving diagnostic value. For the optimization of medical radiation exposures, it is important to establish the DRL for specific clinical circumstances, for example, patient age groups and human body parts under clinical investigations. Up to 50% radiation exposure can be reduced by applying the correctly adjusted exposure parameters, such as changing the tube current and voltage and applying the AEC, ATCM, and ATVS functions. Furthermore, the prefiltering practice, with added filters, and the usage of iterative reconstruction techniques in the course of post-processing not only enhance the image quality but also decrease the radiation dose to patients. Moreover, the integration of AI and deep learning techniques, along with the use of iterative image reconstruction techniques, has the potential to reduce noise from clinical CT images. The review of the literature reveals that the guidance for CT scan operators guarantees pediatric patient safety and high-quality diagnostic images. These approaches not only protect pediatric patients but also increase the overall efficacy of CT abdominal imaging across all age groups.
